# The Chelating Ability of Plant Polyphenols Can Affect Iron Homeostasis and Gut Microbiota

**DOI:** 10.3390/antiox12030630

**Published:** 2023-03-03

**Authors:** Aurelia Scarano, Barbara Laddomada, Federica Blando, Stefania De Santis, Giulio Verna, Marcello Chieppa, Angelo Santino

**Affiliations:** 1 Institute of Science of Food Production, C.N.R. Unit of Lecce, 73100 Lecce, Italy; 2 Department of Pharmacy-Pharmaceutical Science, University of Bari Aldo Moro, 70125 Bari, Italy; 3 Digestive Health Research Institute, Case Western Reserve University School of Medicine, Cleveland, OH 44106, USA; 4 Department of Biological and Environmental Sciences and Technologies (DISTEBA), University of Salento, 73100 Lecce, Italy

**Keywords:** polyphenols, iron metabolism, inflammation, gut microbiota

## Abstract

In the past decades, many studies have widely examined the effects of dietary polyphenols on human health. Polyphenols are well known for their antioxidant properties and for their chelating abilities, by which they can be potentially employed in cases of pathological conditions, such as iron overload. In this review, we have highlighted the chelating abilities of polyphenols, which are due to their structural specific sites, and the differences for each class of polyphenols. We have also explored how the dietary polyphenols and their iron-binding abilities can be important in inflammatory/immunomodulatory responses, with a special focus on the involvement of macrophages and dendritic cells, and how they might contribute to reshape the gut microbiota into a healthy profile. This review also provides evidence that the axes “polyphenol–iron metabolism–inflammatory responses” and “polyphenol–iron availability–gut microbiota” have not been very well explored so far, and the need for further investigation to exploit such a potential to prevent or counteract pathological conditions.

## 1. Introduction

Polyphenols are secondary metabolites that are naturally present in many plant-derived food and beverages that are commonly consumed in the human diet [1,2,3]. According to their chemical structure, polyphenols can be divided into the following different sub-groups: phenolic acids (including hydroxycinnamic and hydroxybenzoic acids, commonly present in coffee, tea, cocoa, oats, rice, wheat, and some fruits); flavonoids, which include different subclasses, such as flavones (reported in vegetables and fruits, e.g., citrus fruits), flavanones (citrus fruits), isoflavones (soya-derived products), flavonols (mostly present in vegetables, such as onions, tomatoes, cauliflower, and broccoli), flavanols (wine, tea, and cocoa), and anthocyanins (berries, grapes, and red wine); stilbenoids (grapes, berries, peanuts, and wine); tannins (grapes and wine); and lignans (seeds and grains) [4,5,6,7].

Polyphenol-enriched diets exert several beneficial roles in human health, thus supporting the prevention of many non-communicable diseases that are associated with oxidative stress and inflammation [8,9,10]. Specifically, these bioactive compounds from foods are able to modulate human health at multiple levels, as follows: acting as scavengers of reactive oxygen species (ROS) [11]; imprinting an anti-inflammatory profile to the innate immune cells; downmodulating inflammatory cytokines and chemokine secretion; inhibiting the correct immunological synapse formation and, consequently, the initiation of antigen-specific adaptive immunity [12,13]; modulating some important cellular pathways that are linked to tumorigenesis, such as cellular proliferation [14]; and regulating gut microbiota [15].

One of the possible mechanisms that could connect all of these biological activities is the iron-chelating ability of polyphenols, which can influence iron homeostasis in the human body. In fact, iron is a pivotal microelement for living organisms. Indeed, upon the onset of an infection, one of the primary host responses is related to iron sequestration and accumulation into phagocytes. Vice versa, tissue repair is characterized by the immune cells’ depletion of the cytoplasmic iron content.

Iron chelation can negatively impact iron deficiency conditions, thus resulting in a higher risk of anemia, which impacts the immune system, also inducing metabolic and neurological impairments [16,17]. On the other hand, iron overload and accumulation can lead to other pathological conditions, such as hemochromatosis, metabolism dysfunction, and other complicated symptoms [18]. Excessive iron availability represents a risk factor for cancer development, as a consequence of the increased level of reactive iron, which promotes ROS and DNA damage and mutations. Therefore, the chelating activity of polyphenols can play a positive role in iron-overload conditions by helping to reverse the damages that are caused by iron accumulation and, consequently, can act as a nutritional tool for chronic inflammatory syndrome prevention. Moreover, many polyphenols can favor the selection of beneficial bacteria that can transform them into molecules with increased anti-inflammatory activity [19]. To date, little is known about the microbiota communities that can contribute to polyphenol–bacteria interaction, but further studies will help us to develop new polyphenol-based prebiotic formulations with important health benefits [20].

## 2. Iron Intake, Absorption, and Homeostasis

Iron is one of the most abundant elements on our planet, constitutive of both Earth’s crust and its inner cores [21]. Living organisms, from prokaryotic to eukaryotic cells, require iron for survival and proliferation. Iron is a constituent of hemoproteins, including those that are involved in oxygen transport and storage (hemoglobin and myoglobin), electron transfer (cytochromes), and iron–sulfur-containing proteins, or proteins which require iron to carry out the essential functions in cellular metabolism [22,23]. The biological importance of iron relies on its chemical properties as a transition metal, since it is involved in redox reactions in both its ferric (Fe^3+^) and ferrous (Fe^2+^) states. However, an excess of reactive iron can be toxic for cells, since it can participate in Fenton-type reactions with the production of hydrogen peroxide, or lipid peroxides, and the generation of ROS. H_2_O_2_ can be further reduced by Fe^2+^ in OH^•^, causing protein modification, nuclear and mitochondrial DNA damage, and the oxidation of other biomolecules, thus triggering genetic mutations and/or cell death. Oxidative stress conditions can also induce iron release from its ligand proteins, resulting in higher concentrations of free iron, which in turn generate an enhanced susceptibility to oxidative DNA damage. Therefore, the level of reactive iron and the balance between cellular iron overload and iron deficiency must be carefully controlled and finely regulated [22].

Most living organisms have developed different strategies to acquire, store, and recycle iron [24]. In plants, two strategies are employed to acquire iron. The first strategy, which is mostly adopted by dicots and non-graminaceous monocots, consists of the acidification of the rhizosphere by excreting protons to reduce Fe^3+^ to Fe^2+^ and the promotion of its uptake. In the second strategy, which is carried out by grasses, rice, maize, barley, and wheat, phytosiderophores (PS) and other high-affinity chelating compounds are secreted to chelate and uptake ferric iron [21].

In humans, iron can be assumed in the form of heme or non-heme from the diet or can be derived from cellular turnover or exfoliation [25]. The dietary sources of heme iron are represented by red meat, liver, fish, and shellfish, whilst non-heme iron is mainly present in pulses and vegetables [24]. Furthermore, the presence of dietary components such as ascorbic acid can promote heme and non-heme iron absorption, whereas other compounds, such as phytic acid, can reduce iron absorption [25,26].

In the human body, almost 60–70% of iron is bound to hemoglobin (Hb), 20% is deposited in ferritin (Ft), and the remaining percentage is linked to both myoglobin in the muscle tissues and to transferrin (Tf) [27].

**Figure 1:**
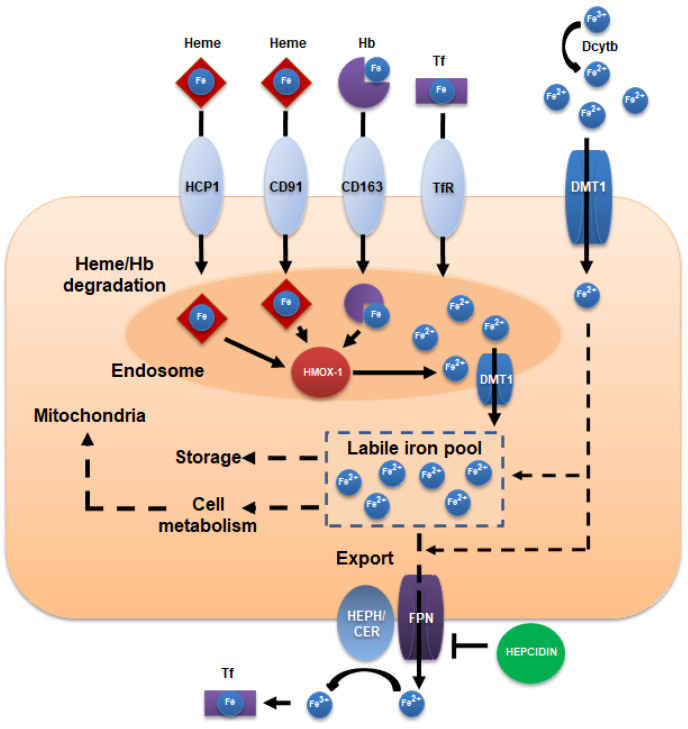
Schematic representation of cellular iron metabolism. Divalent metal transporter 1 (DMT1) mediates the cellular import of Fe^2+^ after its reduction from Fe^3+^ by the cytochrome-b-like ferrireductase (Dctyb). Transferrin receptor (TfR) binds transferrin–iron complexes before their internalization by receptor-mediated endocytosis. DMT1 function is also implicated in the iron transport from the endosome to the cytoplasm, following the Tf cycle. The hemoglobin scavenger receptor (CD163) and heme-hemopexin receptor (CD91) are implicated in hemoglobin uptake. Once inside the cell, iron joins the labile pool that is stored in ferritin or participates in cell metabolism processes. Ferroportin (FPN) mediates the iron efflux outside the cell, mostly under the regulation of hepcidin. Hephaestin (HEPH) or ceruloplasmin (CER) oxidize Fe^2+^ to Fe^3+^ for the binding of iron to transferrin.

Iron absorption mainly occurs at the duodenal level, where the ferrireductase cytochrome b (DcytB) reduces Fe^3+^ to Fe^2+^ in order to allow import by the epithelial divalent metal transporter 1 (DMT1) (Figure 1). Heme iron can be internalized by the low-affinity heme carrier protein HCP1 into the endosome, where it is catabolized by hemeoxygenases (Hmox). Hmox releases free ferrous iron, which is presumably transported to the cytoplasm by divalent metal transporter 1 (DMT1) [22,23]. Here, heme-derived and non-heme iron join the labile iron pool, from which iron can be used by the mitochondria for metabolic reactions, can be incorporated into Ft for cellular storage, or can be exported across the basolateral membrane by ferroportin-1 (FPN-1) into blood circulation. Ferrous iron is subsequently oxidized to ferric iron by the ferroxidases hephaestin, or ceruloplasmin, and bound to Tf [23]. The complex Tf iron interacts with the membrane receptors and is internalized by receptor-mediated endocytosis, which is followed by Tf recycling. Iron uptake also involves the transport of heme–hemopexin complexes, which are internalized via the CD91 receptor (expressed in liver and macrophages), and hemoglobin-bound iron that is complexed with haptoglobin, which is imported via the CD163 receptor (mostly express in monocytes and macrophages).

At a cellular level, iron regulatory proteins (IRP1 and 2) play an important role in the regulation of iron homeostasis by recognizing the iron-responsive elements (IREs) that are located in the untranslated regions of the mRNA encoding proteins that are involved in iron metabolism, such as DMT1, transferrin receptors, Ft, and FPN1, therefore modulating their translation [28]. At a systemic level, an important protein regulating iron homeostasis is represented by hepcidin, synthetized by the liver, which regulates the iron absorption, distribution, and storage in the different body districts [29]. In this way, increased levels of hepcidin reduce iron transport into the bloodstream, therefore affecting the iron distribution and storage, whereas reduced levels of hepcidin can cause an excess of iron in the bloodstream, Tf saturation, iron accumulation and overload, and hemochromatosis [28,30].

## 3. Anti/Pro-Oxidant Activities of Polyphenols

Polyphenols are antioxidant compounds that are well known for their radical scavenging activity. By this mechanism, polyphenols behave as reducing agents towards ROS and reactive nitrogen species (RNS), such as OH^•^, O_2_^•−^, and NO^•^, thus preventing the oxidative stress and DNA damage that are caused by these species. The hydroxyl radical can be generated by different pathways, including the decomposition of peroxynitrous acid [31] or the reduction of peroxides, whereas the production of H_2_O_2_, O_2_^•−^, and NO^•^ is mostly derived by cellular respiration or cell signaling mechanisms [32]. Polyphenols are able to act as antioxidant compounds by donating an electron or hydrogen atom, thus neutralizing the free radicals. They also act as radical scavengers and chain breakers in lipid peroxidation chain reactions, with the consequent formation of more stable and less reactive species and block of chain reactions before the cell viability can be seriously affected [6,33,34]. Furthermore, polyphenols can induce the expression of antioxidant enzymes, such as glutathione peroxidase, catalase, and superoxide dismutase, which act on hydroxyperoxides, hydrogen peroxide, and superoxide anions, and inhibit the expression of pro-oxidant enzymes, such as cyclooxygenases and lipoxygenases [6,35].

Besides their antioxidant abilities, polyphenols have also been shown to behave as pro-oxidant compounds [35,36,37]. In different cases, the pro-oxidant activity has been related to the structural characteristics of polyphenols, e.g., the flavonol quercetin showed a pronounced pro-oxidative activity, while the flavanones hesperetin and naringenin have displayed milder effects [38]. Some flavonoids containing multiple hydroxyl groups, especially in the B-ring, have been shown to increase the production of hydroxyl radicals [39]. Baicalein, containing a pyrogallol structure in the A-ring, has also been reported to promote hydrogen peroxide production [39,40]. In addition, the pro-oxidant effect can be caused by the autoxidation or enzymatic oxidation (for example, by peroxidases) to which polyphenols can be subjected, causing the production of highly reactive phenoxyl radicals, such as flavonoid quinones. These compounds can be stabilized in vivo by conjugation with nucleophiles, such as GSH, cysteine, or nucleic acids [41].

The pro-oxidant activity of polyphenols could also be associated with their ability to reduce Fe^3+^ (or other transition metal ions) and the prevention of their binding to other chelating ligands, such as EDTA. In fact, the pro-oxidant properties of polyphenols have been experimentally observed in the presence of metal chelators, such as EDTA, and the oxidized form of the metal ion Fe^3+^ [36,42,43,44,45]. Increased levels of OH^•^ have been observed following the reduction in Fe^3+^ complexed with EDTA in the presence of myricetin, quercetin, or catechin [42]. Some phenolics (quercetin, phloretin, phloridzin, phloroglucinol, gallic acid, ferulic acid, and 3,4-dihydroxyphenylacetic acid) have been found to enhance OH^•^ generation under the Fe^2+^-EDTA-H_2_O_2_ system [35]. However, an important consideration that should be taken into account is that the intracellular environment is prevalently reducing, due to the presence of reductant agents such as NADH, glutathione, ascorbic acid, etc., [46,47,48]. Therefore, most of the metal ions that are not bound to proteins would be mainly in their reduced forms in vivo [49]. Polyphenols can display both antioxidant and pro-oxidant activities in very similar conditions, even though their pro-oxidant activity may increase in the presence of very strong chelators (such as EDTA, or medications with chelating abilities, e.g., bleomycin) or high concentrations of H_2_O_2_ [35,49]. Furthermore, the pro-oxidative potential of polyphenols can also differ within the same class, dependent on their concentration (low levels of polyphenols may have antioxidant activity, whereas higher concentrations may display pro-oxidant effects), pH conditions, or stereochemistry, which might partly explain the controversy among their antioxidant and pro-oxidant effects [37].

## 4. Iron-Chelating Abilities of Polyphenols

**Figure 2:**
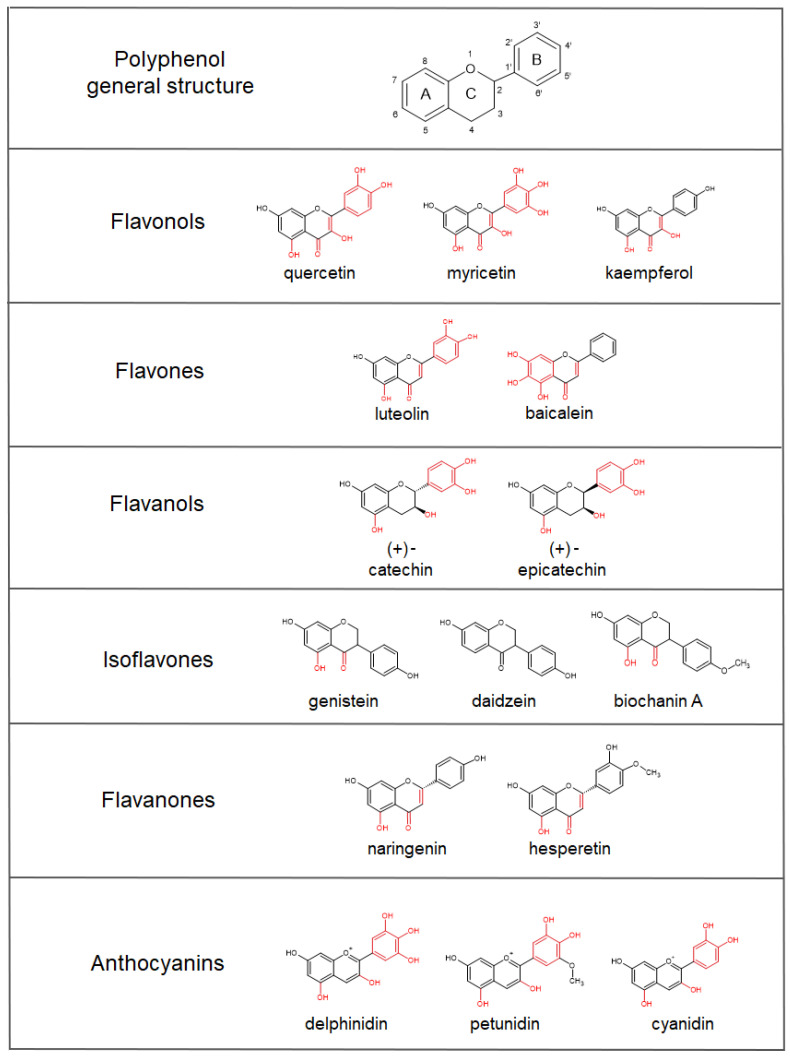

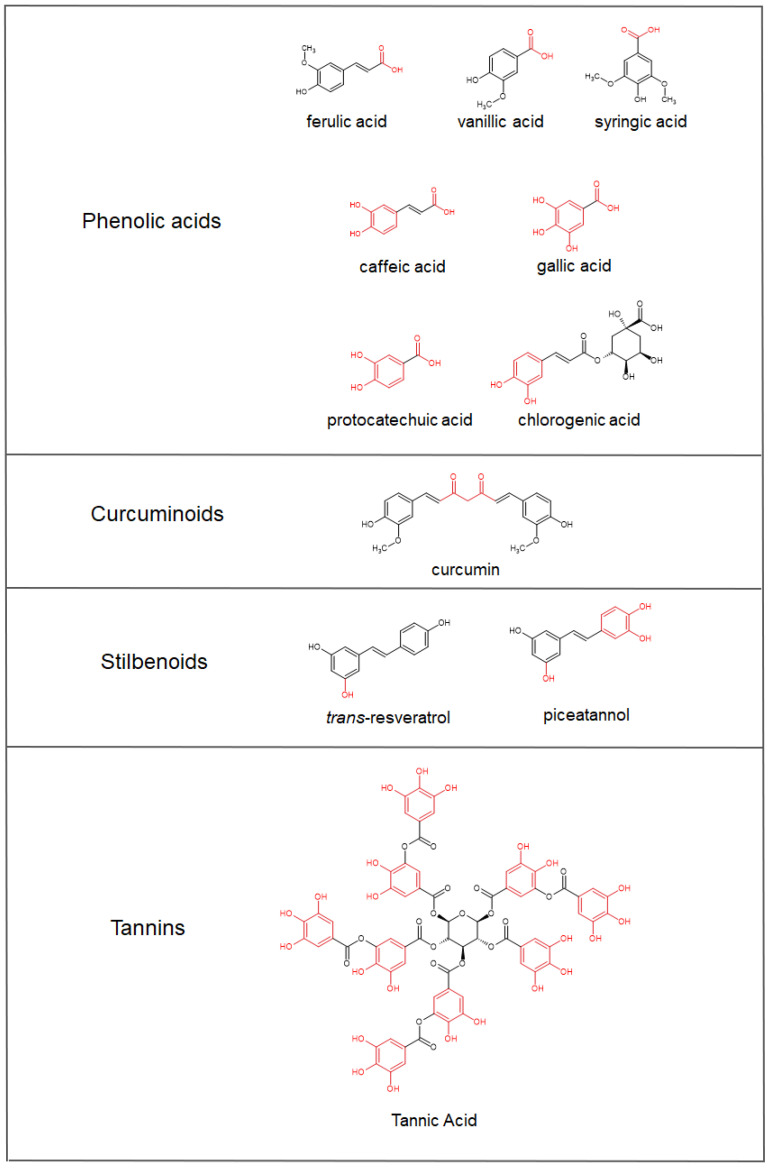
Representative classes of polyphenols and examples of compounds belonging to each group, showing different iron-chelating abilities. Polyphenol general structure is formed by two aromatic rings, indicated as A and B, linked together by three carbon atoms forming an oxygenated heterocycle, the C ring. The 6,7-dihydroxy structure, B-ring catechol, galloyl groups, 2,3-double bond, 3- and 5-hydroxylic groups, β-diketone group, and carboxylic groups associated with iron-binding properties are highlighted in red.

Besides their radical scavenging activities, another mechanism by which polyphenols can exert their antioxidant activity involves iron binding. Iron, and transition metals in general, can be involved in the generation of oxygen free radicals, the reduction of peroxides, or reactions with superoxide anions [50,51,52], and, subsequently, oxidative stress. Polyphenols have been shown to have iron-binding abilities, which are mainly related to the presence of catechol and galloyl groups. Some studies have shown that the 6,7-dihydroxy structure, B-ring catechol, the galloyl groups, the 2,3-double bond, and the 3- and 5-hydroxylic groups in co-presence with the 4-keto group are associated with chelation properties, and therefore are eligible as iron-binding sites [49,53,54,55,56] (Figure 2). For example, baicalein and baicalin, containing 6,7-dihydroxy groups, have strong iron-binding activities [57]. Flavonoids, such as quercetin and rutin, with 3- and 5-hydroxy-4-keto groups, or flavones and flavonols with 2,3-double bonds in general, are also important metal chelators [58,59]. Ellagic acid, with its four hydroxyl groups, shows metal-transition-chelating abilities, with the possibility to participate in antioxidant redox reactions, resulting in an efficient free radical scavenger [60,61]. Interestingly, when it is incubated with iron–EDTA or iron–citrate complexes, ellagic acid is able to remove iron from those ligands by forming an iron–ellagic acid complex, which reduces the levels of iron ions in the solution that catalyze free radical formation, and therefore showing an antioxidant mechanism that is different from “classical” OH^•^ radical scavenging [45]. In the case of curcumin, the β-diketone group has been suggested to be responsible for iron chelation, even though it does not affect or block iron cellular uptake [62,63]. In in vivo systems, curcumin’s iron-chelating abilities show the ability to affect the systemic iron metabolism (e.g., a decline in serum iron and transferrin saturation, decreased iron levels in the spleen and bone marrow, IRPs activation, repressed ferritin levels, and hepcidin hepatic synthesis), thus suggesting possible effects in patients with both a marginal and a high iron status [64,65]. Furthermore, the iron-chelating abilities of curcumin have also been suggested to contribute to anticancer activities through the formation of redox-active iron complexes and iron depletion in cancer cells [63,64,65].

In the case of isoflavones, the 5-hydroxy-4-keto group has been suggested to chelate ferric and ferrous ions, even though the affinity towards these ions was lower than those of the other iron-chelating flavonoids [66]. In particular, genistein and biochanin A, but not daidzein, show chelating abilities of Fe^3+^, indicating that isoflavones bind the metals at the 4-keto and the 5-OH sites [67].

Lakey-Beitia and co-workers [68] propose the following three groups of polyphenols based on the binding sites: a group with one metal binding site, to which belong the curcuminoids, some stilbenoids, isoflavones, and flavanones; the group with two binding sites that includes some flavones and some anthocyanins; and the group with three metal binding sites that includes flavonols, flavanols, some anthocyanins, and tannins.

In general, the polyphenolic compounds with catechol moieties on the B-ring are more potent inhibitors of the Fenton reaction than those without catechol groups [69]. In addition, the presence of a large number of catechol/galloyl groups (as in the case of tannic acid) contributes to enhanced iron chelation. Perron and co-workers [70] have shown that compounds with galloyl groups have a higher antioxidant activity compared to those with only catechol groups. Phenolic acids bearing catechol or galloyl groups (caffeic acid, gallic acid, protocatechuic acid, and chlorogenic acid) have shown more intriguing iron-binding properties compared to the other polyphenols lacking these groups (ferulic acid, syringic acid, and vanillic acid) [53]. For these, the carboxylate group has been proposed as the most eligible group for iron complexation [71]. In addition, the structures with galloyl moiety, as in the case of gallic acid within the group of hydroxybenzoic acids, scored better than those of the catechol type (as in the case of protocatechuic acid), which could be attributed to the number and position of the hydroxyl groups. It is worthy of notice that gallic acid alone exhibits a reduced iron-chelating capacity, which is probably because of the third hydroxyl group in position three. Indeed, this third OH group can stabilize the flavonoid ring structure and has radical scavenging abilities [72,73], but reduces the iron-chelation ability [53]. Similarly, the presence of methoxy groups (as in the case of vanillic acid, syringic acid, and ferulic acid) increases the radical scavenging activities but hinders the chelation abilities of polyphenols [53,74].

## 5. Polyphenols’ Bioavailability

The bioavailability of dietary nutrients and compounds usually designates the quantity or fraction of the ingested dose that is absorbed from the gastrointestinal tract [75]. In the case of polyphenols, the bioavailability is strongly influenced by their physical properties (e.g., molecular mass, polarity, and hydrophobic moieties) and the presence of proteins, lipids, and fibers in the ingested food matrixes [76]. For instance, poor anthocyanin bioavailability can be improved by their binding to dietary fibers, which are able to protect them from degradation due to the pH of the intestinal environment and allow them to reach the large intestine and remain there for a longer time [76]. Once they are ingested, polyphenols reach the intestine, where they are first deconjugated by enzymes such as lactase phlorizin hydrolase (LPH, located on the enterocytes membrane) or β-glucosidase (CBG, cytosolic), in order to facilitate the absorption by the epithelial cells. Following this first process of absorption, the polyphenols are transported through the portal vein to the liver, where they undergo phase I metabolism, which implies hydrolysis and oxidation reactions, and phase II metabolism, prevalently including glucuronidation, methylation, and sulfonation reactions [77,78]. Such metabolites can have markedly changed iron-chelating and anti/pro-oxidant properties [79,80]. They can be detected in systemic circulation; however, such modifications (in particular, sulfation and glucuronidation) also facilitate their efflux into the intestinal lumen. Once they are released into the intestinal lumen, the polyphenols are then subjected to microbial biotransformation, which can produce smaller and structurally simpler compounds [81,82]. Several studies report the presence of phase II metabolites (glucuronides or sulfated forms) in plasma samples after the ingestion of polyphenols, or foods that are enriched in polyphenols, indicating that the intestines and the liver are the main sites of polyphenol metabolism [83], whereas polyphenolic aglycones are not commonly found there [84,85]. Table 1 reports some examples from published studies describing the bioavailability and the excretion paths of representative classes of polyphenols. The maximal plasma concentration (C_max_) and the time to reach it (t_max_) strongly depend on the diverse classes of compounds, the type and amount of the food source, the studied species, and the interindividual variability [86,87]. In some cases, such as for quercetin, the type of bound sugar moiety can also influence the efficiency of the intestinal absorption and, therefore, the polyphenol bioavailability [83].

The main route of elimination of polyphenols is urinary excretion, even though biliary excretion should be considered for some compounds, such as quercetin or curcumin [87,88,89,90].

Polyphenols’ intestinal absorption is mainly mediated by epithelial glucose or monocarboxylates transporters (MCTs) [91]. Quercetin glucosides can be directly transported via SGLT1 (sodium-dependent glucose transporter 1) or hydrolyzed to quercetin before their absorption by passive diffusion in the small intestine [83]. Rutin is metabolized by intestinal bacteria into phenolic compounds prior to being absorbed via MCT, or through the paracellular pathway. The uptake transporters OATPs (organic anion transport polypeptides) and OATs (organic anion transporter) contributes to the uptake of quercetin and its metabolites into the liver and the kidneys [83]. A hesperetin derivative, MTBH (8-methylene-tert-butylamine-3’,5,7-trihydroxy-4’-methoxyflavanone), has been shown to be mainly absorbed by the transcellular passive diffusion mechanism and to use MCT carriers to enter the cells [92]. Trans-resveratrol has been reported to use a passive transport to cross the apical membrane of the intestinal cells, whereas the transport of its trans-piceid derivative is likely to be active, involving SGLT1 [93]. On the other hand, the main transporters that are implicated in polyphenol efflux are MRP2 (multi-drug resistance protein 2), BCPR (breast cancer resistance protein), and P-gp (P-glycoprotein transporters) [91]. MPR2 and BCPR mediate the excretion of quercetin and its metabolites through bile and urine, eliminating them from the body [83]. MRP2 has been also shown to be involved in stilbene efflux [93]. P-gp and BCPR are used for MTBH efflux transport [92]. However, Teng and co-workers (2012) [91] reported different affinities for SLGT1, MRP2, and P-gp transporters among the different classes of polyphenols, indicating that their influx or efflux may be dependent on their chemical structures.

**Table 1:** Examples of studies reporting polyphenols’ bioavailability in humans.

Polyphenol Compound—Food Source	Dosage	C_max_^1^ of the Main Compounds in Plasma (t_max_^2^)	Main Compounds Excreted in Urine (Time)	Reference
Quercetin-4’-O-glucoside	100 mg	2.12 µg·mL^−1^ of quercetin-4’-O-glucoside (0.70 h)	NA **^3^**	[94]
Quercetin-3-O-rutinoside	200 mg	0.32 µg·mL^−1^ of quercetin-3-O-rutinoside (6.98 h)	NA	[94]
Fried onions (containing 275 µmol flavonols, principally quercetin-4’-glucoside and quercetin-3,4’-diglucoside)	270 g	665 nM of quercetin-3’-sulfate (0.75 h) 351 nM of quercetin-3-glucuronide (0.60 h) 63 nM of quercetin diglucuronide (0.80 h)	274 nM quercetin diglucuronide (8–24 h)	[95]
Fresh blackcurrant (containing 897 mg total anthocyanins)	100 g	Anthocyanins not detected	339 µg total anthocyanins (48 h) 17.3 mg hippuric acid (0–24 h)	[84]
Elderberry concentrate (containing 1.9 g anthocyanins and equivalent to 235 mL fresh juice)	11 g	NA	15.8 µg cyanidin-3-glucoside (1 h) 29.8 µg cyanidin-3-sambubioside (2 h)	[96]
Homogenized raspberries (containing 292 µmol anthocyanins, 6.3 µmol ellagic acids, 251 µmol ellagitannins, and 2.5 µmol phenolic acids)	300 g	180 nmol·L^−1^ of 3’,4’-dihydroxyphenylaceticacid (6 h) 78 nmol·L^−1^ of 4’-Hydroxyhippuricacid (1 h) 47 nmol·L^−1^ of ferulic acid-4′-sulfate (1.5 h) 18 nmol·L^−1^ of ferulic acid-4′-O-glucuronide (1.5 h) 14 nmol of isoferulic acid-3’-O-glucuronide (1.5 h)	19.9 nmol cyanidin-3-O-glucoside (0–48 h) 6.4 nmol 4-Hydroxybenzoic acid (0–48 h) 6.5 nmol ferulic acid-4-sulfate (0–48 h) 16.1 nmol 4-hydroxyhippuricacid (0–48 h) 239 nmol hippuric acid (0–48 h)	[97]
Evelor 500 mg tablets (containing trans-resveratrol)	500 g	71.18 mg·mL^−1^ of trans-resveratrol (1.339 h) 1516.014 mg·mL^−1^ of sulfated resveratrol 4083.900 mg·mL^−1^ of glucuronated resveratrol	NA	[98]
Coffee beverage, containing various doses of chlorogenic acid: 412 µmol (A) 635 µmol (B) 795 µmol (C)	200 mL	808 µmol of total chlorogenic acid derivatives (0.5–6 h) (A) 1242 µmol of total chlorogenic acid derivatives (0.5–6 h) (B) 1164 µmol of total chlorogenic acid derivatives (0.5–6 h) (C)	100.7 µmol of total chlorogenic acid derivatives (0–24 h) (A) 160.0 µmol of total chlorogenic acid derivatives (0–24 h) (B) 125.2 µmol of total chlorogenic acid derivatives (0–24 h) (C)	[99]
Curcuminoids as native powder, micronized powder, or liquid micelles (containing 410 mg curcumin, 80 mg demethoxycurcumin, and 10 mg bis-demethoxycurcumin)	500 mg	7.1 nmol curcumin (7.5 h) (native powder) 41.6 nmol curcumin (8.8 h) (micronized powder) 3228 nmol curcumin (1.1 h) (liquid micelles)	5.1 nmol ^4^ curcumin (0–24 h) (native powder) 70.6 nmol ^4^ curcumin (0–24 h) (micronized powder) 753 nmol ^4^ curcumin (0–24 h) (liquid micelles)	[100]

^1^ C_max_: maximal plasma concentration. ^2^ t_max_: time to reach C_max._^3^ NA: not available. ^4^ data expressed as nmol·g^−1^ creatinine.

## 6. Polyphenol-Mediated Iron Sequestration Affects the Inflammatory Response

### 6.1. The Host Level

Polyphenols display remarkable anti-inflammatory and modulatory activities on the immune system, at both the intestinal and the systemic level. Such activities involve cellular mediators—such as macrophages, lymphocytes, and dendritic cells—and protein mediators, such as cytokines and interleukins. Polyphenols can impair the release of interleukins, such as IL1β, IL-6, and IL-8, the tumor necrosis factor (TNF) [101,102,103], and chemokines [12]. They can also affect the signaling systems that are involved in inflammatory processes, as in the case of T-cell proliferation and B lymphocyte activation [34]. Polyphenols can also modulate the NFκB signaling and MAP kinase pathways [104,105] and the production of mediators of inflammation through phospholipase A_2_ and cyclooxygenase 2 (COX-2), which are enzymes that are involved in the arachidonic acid metabolism [106,107,108]. Another important anti-inflammatory mechanism concerns nitric oxide (NO) production at the vascular level, since polyphenols can inhibit NO release, which is involved in the inflammatory responses that are triggered by free radicals [5]. Polyphenols also inhibit extracellular-matrix-degrading enzymes, such as the matrix metalloproteinase-2 and 9 [109,110,111].

Among these important modulation mechanisms on the inflammatory and immune-related pathways, another interesting route concerns cellular iron homeostasis. The iron-chelating properties of polyphenols can play important roles in systemic iron regulation and during the inflammatory processes at the gut level and/or in iron overload conditions, such as hemochromatosis.

Under homeostatic conditions, iron undergoes systemic recycling to supply the needs of the body metabolism. Dietary iron intestinal absorption compensates the body losses only in part (for example, through intestinal epithelium desquamation or menstrual bleeding [112]). Hence, senescent red blood cells (sRBC) represent an important supply of iron for hemeproteins de novo synthesis (e.g., hemoglobin) and erythropoiesis [112]. Heme deriving from sRBC is mainly recovered by macrophages in the red pulp of the spleen, the liver, and the small intestine, and phagocytized and degraded by Hmox-1 to release iron into the cytoplasm, which is subsequently exported by ferroportin. Such iron release from macrophages is important, not only for iron recycling, but also for tissue iron availability in homeostatic and pathological conditions, as well as in wound healing [113]. In this context, liver hepcidin exhibits an important systemic regulatory role, acting as a ferroportin gene expression inhibitor or representing negative feedback for iron release, based on the iron plasmatic concentrations [114]. The systemic release of soluble inflammatory mediators, mainly IL-6, induces the hepatocytes to release hepcidin, which, in turn, downregulates ferroportin activity, resulting in the inhibition of iron release from the cells and iron absorption under inflammatory conditions [115,116].

It is worth noting that iron homeostasis dictates the polarization of innate immune cells, with an indirect effect on the adaptive immune response. This is especially true for macrophages exerting different immune functions, ranging from pathogen recognition, antigen processing, phagocytic clearance, and positive/negative immune regulation in the resolution of the immune response and tissue repair [117]. These immune functions depend on the macrophages’ functional states; in fact, based on the local environment, macrophage plasticity identifies two phenotypes: the M1 macrophage phenotype, or the “classically” activated phenotype, with pro-inflammatory activities, and the M2 macrophage phenotype, or the “alternatively” activated phenotype, with anti-inflammatory activities and tissue repair properties [118]. The two macrophage phenotypes are characterized by marked differences in the effector functions and the gene expression profile. However, the identification of M1 and M2 largely oversimplifies the plasticity of the macrophages, which rather shows a continuum of the functional activation states that are modulated by the environmental stimuli, such as the iron content in a given cell or tissue [119]. Specifically, macrophages represent one of the major sources of the available iron in our body, thus becoming crucial in iron homeostasis regulation. During inflammation, the macrophages sequester iron, while during tissue repair, the macrophages release iron, also contributing to cell proliferation [120]. Furthermore, macrophage polarization could affect the systemic iron balance that modulates the expression of the hepcidin and ferroportin genes [121]. While M1 polarization induces the up-modulation of hepcidin, promoting iron sequestration and retention, M2 polarization upregulates ferroportin gene expression, enhancing iron release [113,122,123].

Moreover, the balance between iron influx/efflux also modulates other important cells of innate immunity, i.e., the dendritic cells (DCs), which are activated to support inflammation, tissue healing, and host tolerance [124]. DCs maturation is inhibited by iron depletion, since inflammation favors iron influx and the tolerance iron efflux [124]. On the other hand, in in vitro iron-overload-mimicking conditions, bone-marrow derived DCs (BMDCs) have been found to differentiate into MHCII^low^CD11c^+^CD11b^+^F4/80^+^ cells, with a reduced ability to release inflammatory cytokines [125].

Another environmental stimulus that is able to influence the polarization of macrophages is diet. The vast majority of immune cells are located in mucosal tissues, and macrophages and DCs in particular patrol the intestinal epithelium from the basolateral side, sometimes projecting dendrites into the intestinal lumen. Thus, it is not surprising that the luminal content can influence the immune cells’ response. In fact, the beneficial role of the numerous bioactive compounds that are obtained from foods in the modulation of the inflammatory response has been widely demonstrated, with a focus on immune cells from the innate response, such as macrophages and dendritic cells [12,13,126,127,128,129,130]. Thus, the link between polyphenols, iron metabolism, and the inflammatory/immune response can easily come up, even if it has not been extensively studied until now. To explain polyphenols’ potential to act on iron homeostasis and inflammatory conditions, we can start to consider different in vitro and in vivo studies that have investigated the effects of polyphenols on iron homeostasis (Table 2). Specifically, in the presence of polyphenols, macrophages change their polarization and, therefore, the iron availability, in order to reduce the inflammatory processes [131,132,133,134,135]. In C57BL/6 mice that were subjected to a high-fat diet, the administration of a purple, red corn extract that was enriched in anthocyanins resulted in the upregulation of M2 markers in the adipose tissue macrophages, with a downregulation of inflammatory mediators and an increased expression of iron-metabolism genes that were associated with an iron storage reduction [132]. In vitro experiments have shown that quercetin reduces ferroptosis by inhibiting M1 macrophage polarization and ameliorating the inflammatory response [134]. Following quercetin exposure, liposaccharide (LPS)-treated BMDCs change their polarization into an M2-like profile, increasing the ferroportin expression level and, subsequently, the iron efflux, therefore reducing their inflammatory abilities [124,125].

Importantly, when investigating the link between dietary polyphenols and iron metabolism, the modulation of the oxidative pathways has to be taken into account. In iron-overloaded rats, curcumin administration reduced iron accumulation in the spleen and the liver and subsequent oxidative stress by increasing the endogenous antioxidant and anti-inflammatory abilities [136], as also reported in Table 2. Myricetin also reduces iron uptake in Caco-2 cells [137] and the iron content in vivo by inhibiting the expression of transferrin receptor 1 (TFR1) in an Alzheimer’s disease mouse model [138]. Baicalein and quercetin attenuate iron-overload lipid peroxidation and protein oxidation in mouse liver injuries [139]. Low dosages of resveratrol have been shown to reduce ineffective erythropoiesis [140]. Such studies therefore suggest that the iron-chelating abilities of polyphenols can be useful in the reduction in iron overload and the consequent oxidative cellular stress, thus indicating a valuable nutritional strategy to prevent hemolytic disorders.

Moreover, in order to investigate the interaction of ubiquitous polyphenols, such as quercetin and related flavonoids with iron supplements, Mazhar and coworkers administered a low-iron diet to female Sprague Dawley rats for 20 days to induce iron-deficiency anemia [141]. After that, a 30-day treatment with 50 mg kg^−1^ ferrous sulfate, combined in an equal ratio of quercetin, quercetagetin, and patuletin, was administered. The combined administration of flavonoids and ferrous sulfate ameliorates some of the hematological parameters and increases the splenic tissue availability of iron, as well as the expression of ferroportin in this animal model of iron-deficiency anemia. Importantly, this effect seems to be variable with alterations in structural features of flavonoids, even though these functional differences need further investigation. It is important to note that the authors have also proposed that the increase in the serum and splenic stores could be based on the formation of a metal-ion–chelate complex with organic ligands and that could improve their transport across the membrane and the absorption. The positive metallic ion charge could be shielded by polyphenols, thus preventing the interaction with the negatively charged layer of mucin, and thereby increasing their lipophilicity and their absorption by the intestinal enterocytes. Finally, the iron–quercetin complex may provide an alternative pathway for iron absorption through the glucose transporter [141].

**Table 2:** Studies reporting the effects of structurally different polyphenols in iron homeostasis, oxidative stress, and inflammatory conditions.

Compound	Model	Effect	Reference
Anthocyanins	C57BL/6 mice fed with a high-fat diet supplemented with purple corn anthocyanins in drinking water	Downregulation of inflammatory mediators, increased expression of iron genes metabolism, and upregulation M2 markers in adipose tissue macrophages	[132]
Quercetin	In vitro RAW 264.7 cells; in vivo lipopolysaccharide (LPS)/ovalbumin (OVA)-induced neutrophilic asthma mouse model	Reduction in ferroptosis and inhibition of M1 macrophage polarization. The effect was ferrostatin-like	[134]
Quercetin	In vitro bone marrow dendritic cells (BMDCs)	Changes in M2-like phenotype, increased expression in ferroportin, and reduction in inflammatory abilities	[124]
Quercetin and baicalin	Kunming mice fed with a diet supplemented with iron-dextran and baicalin 1% or quercetin 1%	Inhibition of iron-overload induced lipid peroxidation and protein oxidation in the liver	[139]
Resveratrol	Kunming mice with iron-overload induced liver fibrosis	Regulation of iron homeostasis by reducing the expression of hepcidin, ferritin, TfR, and DMT1, and raising the expression of FPN-1	[142]
Resveratrol	In vitro human β-thalassemic-erythroid cells; in vivo in β-thalassemic mice (Hbbth3/+)	Reduction in ineffective erythropoiesis, increase in hemoglobin levels, and reduction in oxidative stress in circulating red cells	[140]
Myricetin	In vitro HepG2 cells; HEK293 cells; C57BL/6 mice	Inhibition of hepcidin expression in vitro; and reduced hepatic hepcidin expression, decreased splenic iron levels, and increased serum iron levels in vivo	[143]
Curcumin	C3H/HeNCrl mice fed with different amounts of iron and curcumin	Decreased iron levels in blood, liver, bone marrow, and spleen, induced TfR1, and repressed ferritin and hepcidin	[65]
Quercetin, quercetagetin, and patuletin	Sprague Dawley rats fed with low-iron diet for 20 days, followed by a 30-day treatment with 50 mg kg^−1^ ferrous sulfate supplement combined with quercetin, quercetagetin, and patuletin	Improved hematological parameters and increased splenic tissue availability of iron and ferroportin expression	[141]
Curcumin	In vitro T51B cells	Repression of iron-dependent generation of ROS and inhibition of intracellular iron toxicity	[62]
Curcumin	Sprague Dawley rats receiving iron and curcumin supplementation in drinking water	Reduction in iron overload-induced lipid peroxidation and reduction in oxidative stress in the liver and spleen	[136]
Curcumin	In vitro LLC-PK and NRK52E cells	Stimulation of heme-oxygenase1 expression	[144]

### 6.2. The Gut Microbiota Level

The data that have been obtained from clinical practice indicate that oral iron supplements may have a deleterious effect that is related to inflammatory bowel disease patients (IBD, i.e., ulcerative colitis and Crohn’s disease) with increased intestinal inflammation, thus, intravenous infusion is the recommended strategy [145,146]. This observation can be explained in light of the knowledge that iron availability is an important limiting factor for gut bacterial proliferation, and that less than 20% of orally supplemented iron is absorbed in the duodenum, while the rest becomes available for the intestinal microorganisms. As mentioned previously, the host response to inflammation accounts for numerous strategies that are aimed at limiting iron availability. Among these, an important role is displayed by the iron-chelating Lipocalin 2 (LCN2), which is a protein that is produced by enterocytes, hepatocytes, macrophages, neutrophiles, and myeloid cells that can limit microbial overgrowth.

At the intestinal level, LCN2 is mostly expressed by epithelial and myeloid cells and shows a very strong affinity toward the bacterial siderophores and a strong bacteriostatic function, since its binding to siderophores, such as enterobactin, inhibits iron acquisition by the bacteria, therefore limiting their growth. A lack of LCN2 results in gut dysbiosis in Lcn2^−/−^ mice [147], and, under intestinal inflammation conditions, it facilitates the growth of pathogenic bacteria and a severe colonic inflammation status. LCN2 may therefore shift the microbial community towards a beneficial milieu in the case of chronic intestinal inflammation [148,149]. However, the pathogens that do not strictly rely on enterobactin-mediated iron acquisition can gain an advantage from LCN2-mediated commensal growth [25]. Furthermore, increased levels of LCN2 have also been associated with pro-inflammatory conditions, insulin resistance, and obesity-related disorders [148]. Iron availability at the intestinal level can be affected by both the commensal bacteria and by the dietary components. In fact, with their abilities to capture iron, the commensal bacteria share iron among themselves and with the human host [150,151]. Furthermore, the bacteria can indirectly improve the iron availability by enzymatically degrading the iron chelators, such as tannins or phytates [152]. In addition, dietary components such as ascorbic acid and organic acids (citric acid, malic acid, etc.), both deriving from the diet or as by-products of bacterial metabolism (lactic acid and propionic acid), can improve the iron bioavailability and bio-accessibility [153].

**Figure 3:**
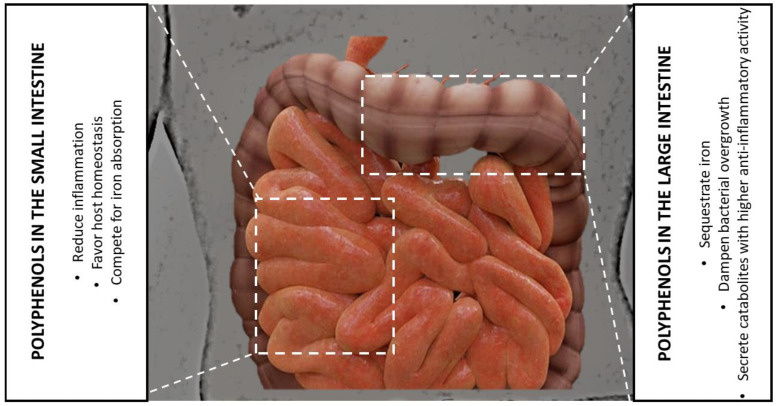
Schematic representation of the polyphenols’ role at the level of the small and large intestines, being important in the reduction in inflammation, iron metabolism, bacterial growth, and metabolite production with anti-inflammatory activities.

Polyphenols, which are characterized by well-known iron-chelating abilities, can play either the role of antimicrobial agents [154] or as growth promoters of some bacterial species [155,156]. This could be also explained by the very limited bioavailability of polyphenols; in fact, while only a small percentage of the total intake is absorbed in the small intestine, the vast majority of it can reach the lumen of the large intestine and can interact with the gut microbiota [157]. Considering the relationship between the polyphenols and iron, DMT1 fails to transport the polyphenol–iron complex into the epithelial cells, and, in this way, the polyphenol–iron complex reduces the iron availability in the intestinal lumen, impairing the gut microbiota growth. This aspect should be considered during intestinal inflammatory events that are associated with microbial dysbiosis, because the iron sequestration that is mediated by iron–polyphenol complexes could be an effective strategy to deprive the gut microbial species of a crucial supply. Furthermore, polyphenols can be the substrate for the growth of some microbiota species, and not others, and the by-products deriving from the microbial metabolism and biotransformation can have effects on the host [5,158] (Figure 3).

In in vitro fermentation and in vivo experiments, increased dietary iron reshapes the gut microbiota composition, with reduced inflammatory responses, increased toxic metabolites, and bacterial-virulence-associated pathways. Such changes therefore predispose the system to an increased risk of infections, the development of a dysbiotic phenotype, and a predisposition to disease [159,160]. Commensal species, such as lactobacilli and *Bifidobacterium*, have been shown to have lower iron requirements compared to other pathogenic strains belonging to *Enterobacteriaceae* (e.g., *Salmonella*), *Yersinia*, *Pseudonomas*, *E. coli*, and *Clostridium* (e.g., *C. difficile*) [161,162]. Some lactobacilli species, such as *Lactobacillus plantarum*, have been found to be able to grow in iron-restricted media [163]. *Lactobacillus sakei* has shown a complete machinery for iron sequestration and metabolism but does not require complete dependence on iron to grow; although, the iron sources enhance its survival rate [164]. Some *Bifidobacteria* strains antagonize *Salmonella Typhimurium* and pathogenic *E. coli* in vitro by sequestering and subtracting the iron from their growth [165]. The non-pathogenic *E. coli* strain Nissle utilizes an LCN2-resistant salmochelin to acquire iron and in such a way that it is able to compete with *Salmonella* [166,167]. Despite these observations, the studies on the changes in the composition of gut microbiota following iron supplementation are insufficient and do not allow for whole generalization. In many studies, iron supplementation has been reported to either to increase, reduce, or have no effect on the different microbiota genera [168,169,170], according to the previously used models. However, consistent data are available to date regarding mostly *Lactobacillaceae* and *Bifidobacteriaceae*, which decrease upon iron supplementation [159,160,169,171]. In this context, the presence of polyphenols in the gut environment could change the iron availability for the gut bacterial species; although, in an iron-deprived environment, some pathogenic strains producing siderophores could benefit and scavenge from the polyphenol–iron complexes [171]. Apart from this possibility, polyphenols may act to prevent the iron uptake of bacteria, therefore dampening the overgrowth of the intestinal bacteria. This could be particularly important in the case of inflammatory conditions that are affected by dysbiosis, as in IBD; however, future studies will be required in order to understand if a polyphenol-enriched diet may be integrated with probiotic supplements to better support the growth of the correct microbiota. In this regard, it is important to underline that microbial metabolites have a crucial role in intestinal pathogenesis; however, recent evidence has shown that they are also involved in host iron absorption and metabolism. Butyrate, diaminopropionate, and reuterin can act on the duodenal enterocytes to reduce iron absorption through HIF-2α activity. Moreover, these metabolites reduce the expression of intracellular iron storage proteins, such as ferritin. These metabolites are known to be produced by bacteria of the *Lactobacilli* genus, and others, and are part of the short-chain fatty acids (SCFAs) class. Thus, they can have a dual role in the host. They can reduce and modulate excessive inflammatory responses and, at the same time, affect iron absorption. Interestingly, this can potentially be employed in probiotic formulations that are used to treat iron overload, or in hemochromatosis to reduce body iron concentrations and excessive inflammation [172].

Iron might have a role in the modulating effects of bacterial-derived SCFAs [173]. The use of an iron-deficient diet in rats reduced butyrate- and propionate-producing bacteria compared to the control animals [174], which might be due to the need for iron by the enzymes that are involved in SCFAs synthesis. Even though the iron levels need to be kept low in order to not incur excessive inflammation, it is also necessary to feed enough of this microelement to the “good” bacteria, since they can also chelate it and reduce its availability to the pathogenic species. *Streptococcaceae*, *Pesteurellaceae,* and *Methanobacteriaceae* were found to be involved in the metabolism of histidine from imidazole propionate, and this, in turn, impairs the insulin signaling in the liver and the adipose tissue [175].

In general, iron and microbiota share a role in modulating the host’s immune system and physiology; microbes fight over iron ions to grow, stealing them from each other and from the enterocytes, while becoming deprived of the host through LCN2 and lactoferrin. While an excess in iron concentration can favor the growth of pathogenic bacteria and induce the onset of several diseases, the right amount of this ion is necessary for eubiotic bacteria for their production of metabolites that are beneficial to the host. In this interplay, polyphenols intervene to chelate more iron and reduce its bioavailability to the pathobionts. The iron is then metabolized by other bacteria that produce health-promoting molecules.

## 7. Polyphenols as Prebiotics—Chelating Iron to Select Beneficial Bacteria

The iron-chelating activity of polyphenols shapes the intestinal microbiota and reduces dysbiosis. The new populations that arise are beneficial to the host, as they can further boost the polyphenol anti-inflammatory effects through the production of polyphenol derivatives [176]. An example of this is the production of bacteria-derived metabolites, e.g., succinate by some genera of *Lachnoclostridium*, and this process has shown positive improvements in the healing of inflamed mucosa; additionally, polyunsaturated fatty acids can be produced by the intestinal bacteria [177]. These bacteria genera were often seen to increase after treatment with polyphenol-enriched diets.

To this extent, polyphenols and iron-chelating molecules can be employed as prebiotics, using their iron-depriving ability to reduce pathogenic bacteria, while, at the same time, favoring the thriving of beneficial species that use the unabsorbed polyphenols to produce other anti-inflammatory compounds [178].

Over time, many bacteria genera have been studied in order to observe how they metabolize polyphenols and what they can produce. *Lactobacilli* can use mulberry polyphenols to produce chlorogenic, caffeic, and ferulic acid metabolites [179]; *Bacteroides* can convert rutin to quercetin [180]; and *Bifidobacteria* can also use sea buckthorn to synthetize caffeic acid [181]. Firmicutes of the *Eubacterium*, *Clostridium,* and *Flavonifractor* genera can metabolize flavonols to obtain SCFAs and their derivatives [182]. Many other bacteria genera have already been tested in vitro in order to observe what pathways are involved in the metabolism of polyphenols and what enzymes could be employed directly to convert the natural molecules into more powerful bioactive compounds. This research area is critical in the production of new, healthier prebiotic formulations that can be commercialized in the next years [183,184]. Other bacteria of the *Akkermansia* genus were found to be able to metabolize anthocyanins and increase insulin sensitivity [185], and fecal microbial enzymes are able to catalyze polyphenol digestion. Bacteria can break phenolic rings and demethylate and dehydroxylate polyphenols in order to obtain smaller acids and aldehydes.

Furthermore, the gut microbiota transforms ellagic acid into urolithins [186]. They can also act on dietary lignans to produce molecules such as enterodiol and enterolactone through a series of reactions that are orchestrated by several bacterial communities [187]. While this is only the tip of the iceberg, new studies are needed in order to discover more bacteria, ideally at a species or strain level, that can transform and boost polyphenol activity and suppress dysregulated inflammation.

Finally, polyphenols’ anti-inflammatory role has been demonstrated by numerous reports, with most of them reporting their ability to suppress IL-6 secretion. The axis between IL-6 [129,130,188,189] and the hepatic production of several iron-related acute-phase proteins has been previously discussed [29]. Thus, polyphenols may have a dual role, supporting homeostasis during healthy periods and dampening inflammation and dysbiosis during chronic inflammation.

## 8. Conclusions

The iron-chelating abilities of polyphenols can help to explain, at least in part, their antioxidant and anti-inflammatory properties. Such iron-binding properties can also be involved in anti-tumorigenic activities, since polyphenols can reduce the iron availability for cancer cells and have cytotoxic activity towards cancer cells by behaving as pro-oxidants through iron–Fenton reactions [63,65,190,191,192]. Although the presence of specific chelating sites in the polyphenol structure has been reported to be important to explicate their iron-chelation activity, we should take into account that these phytochemicals are deeply transformed (i.e., glycosylated, methylated, acylated, hydroxylated, etc.) before their storage in the plant tissues. Therefore, the iron-chelating properties require in vivo conditions that are favorable to set free the chelating sites. Our need of further studies to clarify the axis between polyphenol–iron-metabolism-inflammatory mediators, and the interplay between polyphenols, iron availability, and microbiota, is clear from this review. Such studies are complicated by the study design and the data interpretation since they are strongly dependent from the adopted model, the iron status, and the iron form (non-heme/heme), and because the interaction between the host, the iron metabolism, and the bacterial iron regulation is not always predictable. Targeting bacteria-specific iron uptake, promoting commensal bacteria, and optimizing iron availability for the host may improve therapeutic and nutritional approaches in the context of gut inflammatory diseases.

## Data Availability

Data sharing not applicable.
